# Incredible Role of Osmotic Adjustment in Grain Yield Sustainability under Water Scarcity Conditions in Wheat (*Triticum aestivum* L.)

**DOI:** 10.3390/plants9091208

**Published:** 2020-09-15

**Authors:** Tahir Mahmood, Muhammad Abdullah, Sunny Ahmar, Muhammad Yasir, Muhammad Shahid Iqbal, Muhmmad Yasir, Shoaib Ur Rehman, Sulaiman Ahmed, Rashid Mehmood Rana, Abdul Ghafoor, Muhammad Kausar Nawaz Shah, Xiongming Du, Freddy Mora-Poblete

**Affiliations:** 1 Department of Plant Breeding and Genetics, Pir Mehar Ali Shah Arid Agriculture University, Rawalpindi 46000, Pakistan; tahirmtaha@hotmail.com (T.M.); 11816098@zju.edu.cn (M.A.); sunny.ahmar@yahoo.com (S.A.); yasirwahla20@gmail.com (M.Y.); shahidkooria@gmail.com (M.S.I.); rashid_cabb@hotmail.com (R.M.R.); 2 State Key Laboratory of Cotton Biology, Institute of Cotton Research, Chinese Academy of Agricultural Sciences, Anyang (CAAS), Anyang 455000, China; 2017y90100037@caas.cn; 3 Crop Science Institute, Agronomy Department, College of Agriculture and Biotechnology, Zhejiang University, Hangzhou 310029, China; 4 Ayub Agricultural Research Institute Faisalabad, Cotton Research Institute, Multan 60000, Pakistan; 5 Institute of Plant Breeding and Biotechnology Muhammad Nawaz Shareef University of Agriculture, Multan 60000, Pakistan; shoaib.rehman@mnsuam.edu.pk; 6 National Key Laboratory of Plant Molecular Genetics, CAS Center for Excellence in Molecular Plant Sciences, Institute of Plant Physiology and Ecology, Shanghai Institutes for Biological Sciences, Chinese Academy of Sciences, Shanghai 200032, China; suleman_uaf@yahoo.com; 7 Pakistan Agricultural Research Council (PARC), Islamabad 44000, Pakistan; ghafoor59pk@yahoo.com; 8 Institute of Biological Sciences, University of Talca, Talca 3460000, Chile

**Keywords:** drought tolerance, osmotic adjustment, physiological and morphological attributes, grain weight, yield sustainability, screening tools, wheat (*Triticum aestivum* L.)

## Abstract

Interrogations of local germplasm and landraces can offer a foundation and genetic basis for drought tolerance in wheat. Potential of drought tolerance in a panel of 30 wheat genotypes including varieties, local landraces, and wild crosses were explored under drought stress (DS) and well-watered (WW) conditions. Considerable variation for an osmotic adjustment (OA) and yield components, coupled with genotype and environment interaction was observed, which indicates the differential potential of wheat genotypes under both conditions. Reduction in yield per plant (YP), thousand kernel weight (TKW), and induction of OA was detected. Correlation analysis revealed a strong positive association of YP with directly contributing yield components under both environments, indicating the impotence of these traits as a selection-criteria for the screening of drought-tolerant genotypes for drylands worldwide. Subsequently, the association of OA with TKW which contributes directly to YP, indicates that wheat attains OA to extract more water from the soil under low water-potential. Genotypes including WC-4, WC-8 and LLR-29 showed more TKW under both conditions, among them; LLR-29 also has maximum OA and batter yield comparatively. Result provides insight into the role of OA in plant yield sustainability under DS. In this study, we figure out the concept of OA and its incredible role in sustainable plant yield in wheat.

## 1. Introduction

A major aspect of wheat breeding programs is the assessment of individual genotypes for agronomic traits across a range of agro-ecological conditions. Plant growth and development is limited by diverse environmental constraints including drought, extreme temperatures, and waterlogging conditions. Breeding for stress-resilient crop varieties has prime importance for sustainable productivity and food security. This is the case of wheat (*Triticum aestivum* L.), which is a staple food of more than 4.5 billion people of the world [1], supplying nearly half of their energy intake [2]. Improving wheat grain yield is a challenging task to meet the population surge in the face of climate change. Therefore, there is a dire need for breeding higher grain yield under harsh environmental conditions.

Prior to plant yield, drought stress affects physiological and morphological traits in plants throughout their life cycle. Plant stability under water deficit conditions is largely dependent on reduced water losses under harsh environments [3,4,5]. Progress in drought tolerance breeding is not satisfactory due to the complexity of measuring drought tolerance, which limits the significant yield improvement. There is no direct approach available to screen genotypes for drought stress tolerance in wheat or any other crop. Therefore, screening and exploiting local germplasms for physio-morphological attributes is a gateway to overcome this environmental constraint [6,7,8].

The utilization of diverse genetic resources has paramount importance in the genetic improvement of wheat, as it results in increasing grain yield potential. The potential for improvement through breeding and selection depends upon genetic diversity existing among genotypes of a breeding population in terms of their responses to stress factors [9]. Physiological attributes, i.e., osmotic adjustment (OA), and other important parameters, can be enhanced through selection and breeding [10]. It has been considered that genotypes having higher OA gives higher yield as compared to having low OA capacity under drought stress. Interestingly, a positive correlation has been observed among plant yield and OA in wheat [11,12,13]. Therefore, OA is a key indicator to screen the wheat genotypes against drought stress. In this regard, various studies of OA association with plant yield have been reported, which are summarized in Table 1.

**Figure 1:**
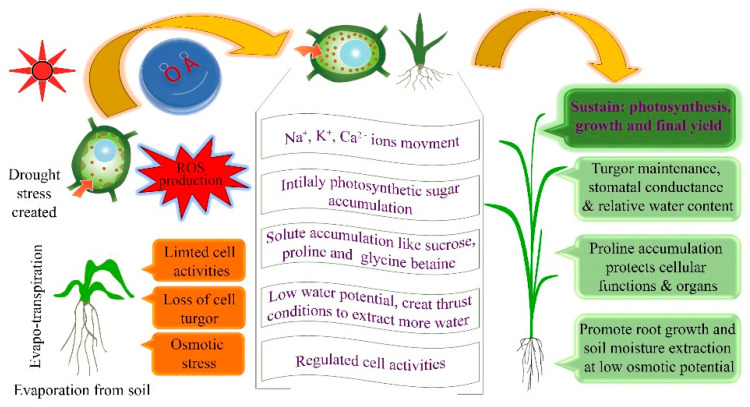
Schematic concept of osmotic adjustment (OA) in plants: how drought coerces to plants for osmotic adjustment under drought stress. Under osmotic stress, plants lose their turgor pressure and water potential, the situation limits the cell activities, and overproduction of reactive oxygen species (ROS) which stimulate the accumulation of water-soluble components for OA. Osmotic adjustment promotes root growth and water extraction from the soil. It maintains the stomatal conductance and turgor pressure to restore the cell activities, photosynthesis, and finally improved plant growth and yield.

After consideration of the relationship of OA among yield and other morpho-physiological traits, the next step is to know how OA conserves water balance and contributes to plant yield under osmotic stress (Figure 1).

Here we have concluded the concept of OA from published studies which will help to understand how and why OA affects plant growth and yield. Recently, our research group has illustrated the association of OA with various physiological and seedling traits in the same panel of wheat genotypes [15]. Plants attain OA through the accumulation of various solutes [24] including total soluble sugars, organic solutes, inorganic solutes or osmolytes, total free amino acids, glycine betaine, proline [25,26], sodium, potassium, and chloride [27,28]. Osmotic adjustment delays leaf rolling prevents cell death [29], and maintains grain yield in crops under water-limited conditions. In multiple ways, OA is involved in sustaining the yield under drought stress environment. Plants attain OA to conserve higher relative water content (RWC) to meet transpiration demands [17] and ensure the cellular membrane stability to control the electrolyte leakage under drought stress environment [30]. Finally, these characters are subsidized for sustained photosynthesis [31] and growth deficit, which finally helps to improve the grain yield in wheat (Figure 1).

The first step in wheat breeding is the comprehensive exploitation of local germplasm and the exploration of genetic diversity. Knowledge about genetic diversity among wheat breeding materials could be instrumental in making breeding strategies [32]. This allows plant breeders to select superior genotypes and to incorporate elite genotypes in breeding programs. In this regard, the main objectives of this study were (i) to investigate physio-morphological traits in local material to unravel drought stress responses (ii) to identify the role of osmotic adjustment with yield and yield component traits in local germplasm and, (iii) to select superior genotypes for breeding improved tolerance to drought stress.

## 2. Results

### 2.1. Analysis of Variance and Mean Performance

Analysis of variance showed significant (*p <* 0.05) effects of the environment, genotype, and genotype × environment interaction for all traits with some exceptions (Table 2). Performance of LLR-29 was noticeable among all selected drought-tolerant genotypes, having maximum thousand kernel weight (TKW), OA, and yield per plant (YP) was also all-out under drought stress (DS) condition. While “Blue Silver” was a consistently high-yielding cultivar under both environments with a lower (0.57) drought susceptibility index for thousand kernel weight (DSI_TKW_). The summary statistics (Table 3) and mean performance of 30 wheat genotypes assessed under well-watered (WW) and DS environmental conditions along with drought susceptibility index for plant yield (DSI_TKW_) are given in Supplementary Table S2.

### 2.2. Principal Component and Biplot Analyses

**Figure 2:**
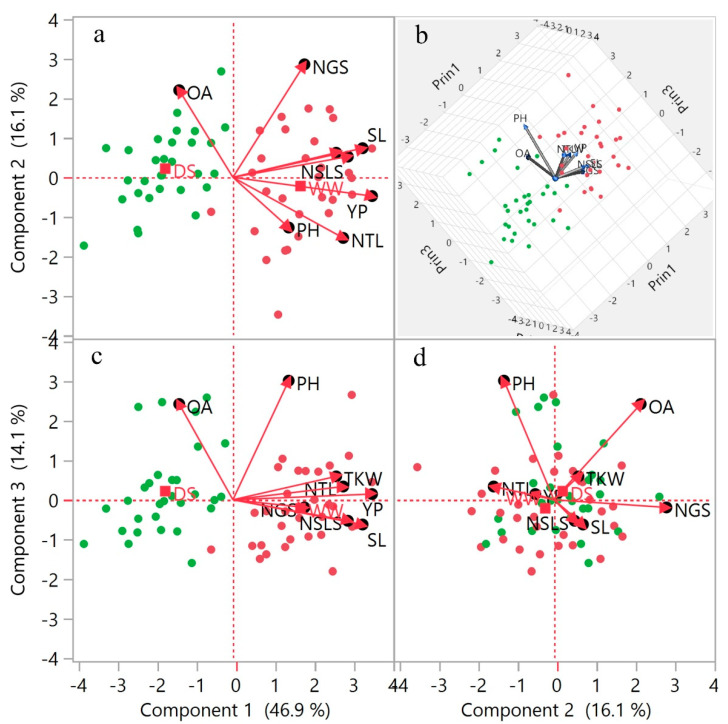
Biplot (Principal component analysis) for all traits assessed in wheat genotypes under well-watered (WW) and drought stress (DS) conditions. Scatter-plot shows the distribution of 30 wheat genotypes for OA, yield and yield components according to principal components 1 and 2 (**a**), components 1 and 3 (**c**), components 2 and 3 (**d**), and 3D scatter-plot of components 1, 2 and 3 (**b**). Here red dots represent genotypes under irrigation and green dots under stress conditions. For trait variables OA, osmotic adjustment; PH, plant height; NTL, number of tillers; SL, spike length; NSLS, number of spikelets per spike NGS, number of grains per spike; TKW, thousand kernel weight, and YP, yield per plant. Cubic Clustering Criterion is given in Supplementary Table S3.

A multivariate, principal component analysis (PCA) biplot was conducted to investigate variability, the response of wheat genotypes toward drought stress, and association among traits and accessions. Variability of traits was explained for principal components 1 and 2, components 1 and 3, components 2 and 3, and 3D scatter-plot of components 1, 2, and 3 in both WW and DS environments. Biplot indicating overall high variability (63%) of components 1 and 3, and (61%) of components 1 and 3. While components 2 and 3 explained very low (32%) variability, which reflects that the first two components are more important (Figure 2). Vector magnitude of OA, plant height (PH), and YP clarified more variability relative to the rest of the traits under both environments, which enhanced the importance of these candidate traits for selection of wheat genotypes under drought stress. Drought stress has a significant effect on OA and gives a large vector than the other yield contributing traits except for PH in biplot analysis, which suggested that plant drifts for OA under the DS environment. Under drought stress condition, five genotypes including WC-4, WC-8, WC-22, LLR-39, and Blue Silver, showed positive and high responses to OA. Most of the genotypes remain stable for yield and yield component traits under stress conditions and fall beyond the yield traits vectors in biplot analysis. Few genotypes including WC-4, WC-22, LLR-14, LLR-29, and Blue Silver are influenced for OA under DS and TKW under WW, so they performed well under both conditions. Genotypes Shahkar-95, Punjab-96, and Kohistan-97 performed well for YP under WW condition butt poorly performed for yield under the DS condition. WC-1, WC-3, LLR-4, LLR-13, and Sehar-06 were the genotypes with the poorest performance under both conditions and stand drought susceptible genotypes. See Supplementary Figure S1 and S2 for more detailed distribution of genotypes with tags and overall PCA summary.

### 2.3. Association among OA, DSI_TKW_, Yield and Yield Components

**Figure 3:**
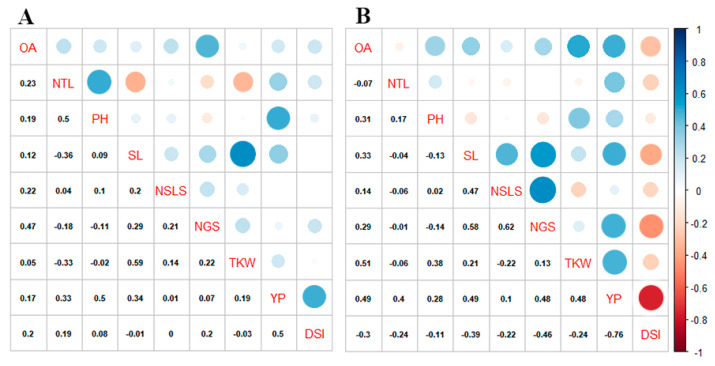
Correlation matrix between OA and yield traits. Left triangle (**A**) for WW and right (**B**) triangle for DS environment.

**Figure 4:**
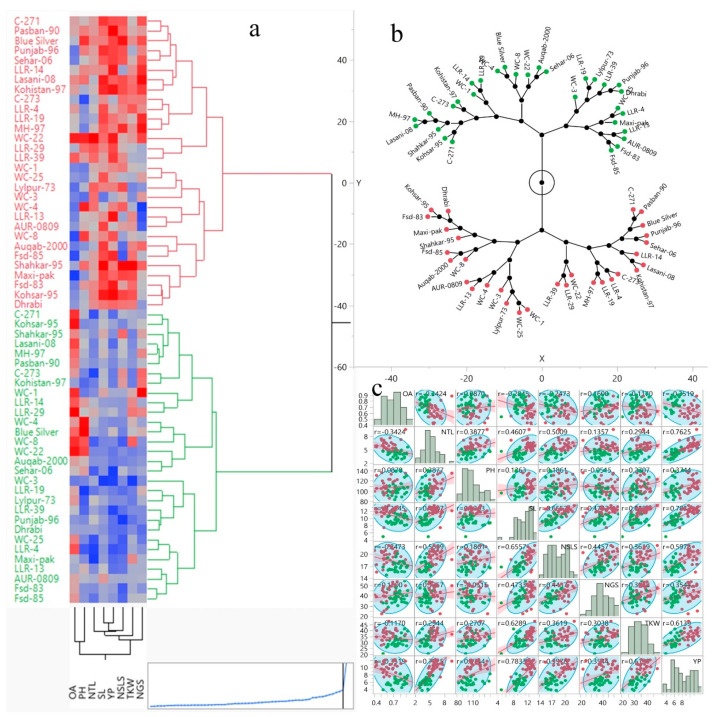
Hierarchical Cluster (**a**), Constellation Plot (**b**) and overall correlation matrix (**c**) of 30 wheat genotypes assessed under WW and DS conditions. Genotypes are distributed in three major and two minor groups in the WW condition. In contrast, genotypes evaluated under DS condition are distributed in four major and 5 minor groups. Here, red color denoted to WW and green to DS. The major and minor groups are classified as having genotypes >6 and >3 respectively.

Osmotic adjustment displayed a highly significant positive correlation among TKW and YP, but non-significant positive relation with the rest of the traits except DSI_TKW_ and number of tillers (NTL). OA negatively correlated with the number of grains per spike (NTL) and DSI_TKW_ under the drought stress environment. While under WW condition OA showed non-significant correlation with most of the traits except NTL. Among the yield components, TKW presented a significant positive correlation with YP and PH including OA, while it had a negative association with DSI_TKW_ under DS condition. On the other hand, under WW condition a negative correlation was observed among NTL, PH, and DSI_TKW_ with TKW. Yield per plant exhibited a significant positive correlation with most of the yield components including number of grains per spike NGS, SL, NTL, and TKW and also correlated with OA; a significant negative correlation was found with DSI_TKW_ under DS condition. Meanwhile, a significant positive correlation was observed between DSI_TKW_ and YP under the WW condition. Most of the traits showed a negative association with DSI_TKW_ under DS condition (Figure 3). Overall correlation matrix among the all traits have given in Figure 4c.

### 2.4. Variability and Diversity Estimation Analysis.

In this study, ridge regression analysis was applied to estimate regression coefficients in the presence of multicollinearity, i.e., a situation of highly intercorrelated independent predictor variables. There are several approaches to select the ridge parameter values in ridge regression analysis. Ridge values suggested being determined from the ridge trace values (Supplementary Figure S3), where a stable set of regression coefficients was attained in graphical options [33]. In Figure 3, the ridge trace showed the values of the ridge parameter from 0 to 0.5, displaying the curves asymptotically, and parallel to the *X*-axis for all parameters. The values were estimated at points 0.4 and 0.3 for DS and WW conditions respectively according to Hoerl and Kennard [34]. Estimated ridge regression coefficients were obtained at the selected values of the ridge parameter for both environmental conditions. The ridge regression analysis indicated 54.54% variability of TKW explained by the seven yield components under the DS environment while only 37.69% variability was observed under the WW environment (Table 4).

There are several available analytical ways to estimate diversity among the germplasm under contrasting environments. We applied Hierarchical Cluster analysis, Constellation Plot analysis, and densities composition plot analysis for wheat genotypes under WW and DS conditions (Figure 4). As shown in Figure 4, there are two distinct classifications of groups under WW and DS conditions. These analyses provided a clearer distribution of genotypes among different environments and within the same environments have different groups. The results indicated that a huge variability is present among the studied genotypes and differential response to the contrasting environments. Genotypes are distributed in three major and two minor groups in the WW condition. On the other hand, genotypes under the DS condition are distributed in four major and 5 minor groups.

## 3. Discussion

Implementation of environmental variables like drought is quite challenging under the field conditions in-regards to avoiding the other environmental effects such as rainfall and sunlight; although they cannot be controlled but can be minimized through little efforts. We made a special structure of tunnel covered with polythene sheet but open from sides to provide a natural environment but controlled irrigation to applied drought stress. We have explored natural diversity of drought tolerance to figure out the effects of drought stress on yield and its components, additionally the role of OA for sustainability of yield under drought environment.

### 3.1. Variability and Effects of Drought Stress on Wheat Genotypes

Previously, just a few studies have been published on Pakistani wheat genotypes and local landraces. Pakistan has a wide diversity in local wheat germplasm, which has been underexplored. Here we conducted an experiment to screen and explore genetic diversity among a panel of 30 wheat genotypes as local germplasm for drought tolerance. Recently, genetic diversity and variation for drought tolerance of wheat have been explored by [35,36]. In this regard, the results of the present study indicated a sustainable genetic diversity in the research material for OA, yield, and yield components, which can be utilized for future breeding programs. The MS values of treatments also indicated the significant results for all the traits (Table 2). Substantially, these results showed that WW and DS treatments have positive effects on all traits, which indicate the existence of a huge variation among the genotypes over contrasting environmental conditions. Our results were consistent with recent studies [35,37] regarding genetic variation and genotype x environment interactions for stress tolerance. The ridge regression analysis indicated a significantly higher variability (54.54%) for TKW explained by the seven traits including YP and yield components under DS condition as compared to the WW environment (Table 3). The significant variation was also directed by principal component analysis under DS condition. High positive loading of NTL, SL, NSLS, TKW, and PY indicates that these traits have more influence and can be simultaneously selected because of their direct influence among each other in the F1 principal component (Table 3) [38]. The response of selected tolerant genotypes toward OA was noticeable in biplot which is clearer in Figure 2b,d; it also supports our selection criteria. Distribution of genotypes among various groups in the Hierarchical Cluster and Constellation Plot analysis indicated a huge variability and differential response of the genotypes to the different environments.

Previously, yield per plant and kernel weight has been employed as a direct selection criterion for improved yield in wheat. However, improvement for stress tolerance appears difficult through natural selection due to the low heritability of YP under stress environments [37]. Drought susceptibility index can help to screen more stable genotypes for drought tolerance. It has been applied to measure the stress tolerance in wheat as a useful tool, for heat stress [37] and drought stress [39]. Drought tolerant genotypes, identified on the bases of DSI (Supplementary Table S2), also showed response toward OA in biplot analysis (Figure 2). This indicates the key role that OA plays in drought tolerance and yield sustainability under a DS environment.

Wheat genotypes respond differently to both contrasting environments. In fact, the reduction in YP, TKW, and other yield components was observed under DS. A significant genotype and environment interaction were also present. Moreover, a significant reduction in yield and its components was observed under drought stress, which also has been reported in previous studies [36,39]. Increased OA was detected under DS as compared to WW condition in our experiment, which indicated that wheat plants accumulate cell solutes under low water potential for OA to extract more water from the soil under the DS environment (Figure 1) [40]. Osmotic adjustment sustains plant yield by interacting with other physiological and morphological traits under drought stress environment. Variability in wheat physiological traits associated with YP, reduction in photosynthetic activity attributed to yield reduction, due to the less amount of assimilates which also affects seed growth [41], and in present case reduction in TKW was observed under DS condition. Interestingly in some genotypes like WC-4, WC-8 and LLR-29 more TKW was observed under DS than the WW condition, among them LLR-29 also have maximum OA and batter yield comparatively (Supplementary Table S2). These results indicate the importance of the OA for sustainable yield under drought. Wheat acclimatizes itself under DS condition through OA for growth recovery after experiencing the stress. Additionally, plants restore their growth and carbon assimilation in new young leaves, which show more tolerance than older leaves [42].

### 3.2. Association of Yield with Osmotic Adjustment and Other Yield Components

Yield per plant has a positive association with kernel weight, spike length, and numbers of grains in a spike, which indicate these components directly contributed to grain yield in wheat under both DS and WW environments. These are the important candidate traits for the selection of drought-tolerant genotypes for dry land and arid areas of the world. In WW condition PH have a strong positive relation with yield, it may be due to more biomass production, stem carbohydrates accumulation. While under DS environment PH contributes to TKW and indirectly associated with plant yield. Accumulation of water-soluble carbohydrate attributes for higher YP followed by a significant increase in TKW in synthetic wheat, was also quantified by Mariano et al. [43]. It has been conveyed that enhanced TKW could be a selection tool for higher YP in wheat. In the present study, TKW was non-significantly and negatively correlated with NGS and NSLS respectively, which indicates that both NGS and NSLS contributed independently to the YP under DS conditions. The reason may be due to less dry matter assimilation ability of single grain during the grain filling period under limited availability of resources [44]. The ridge regression analysis indicated a significant variability (54.54%) for TKW explained by the seven yield components under the DS environment. In our results, PH was positively associated with OA, which indicates that the accumulation of solutes and higher biomass also promotes OA in wheat under the DS environment. A significant positive correlation between OA and biomass was reported by Blum et al. [18]. Subsequently, the association of OA with TKW and SL contributes to the final yield under DS conditions. Perhaps, under stress conditions, the utilization rate of stem stocks during the grain-filled period may contribute to more kernel weight and plant yield, which is also explained by Leport et al. [45]. A positive correlation among OA and NGS was observed which directly contributed to higher YP. More number of grains per spike may the result of higher pollen-viability due to the OA under DS. Recently, the mechanism of OA and maintenance of turgor pressure in wheat pollen grain have disclosed by Khlebova et al. [46], which enhance the confidence of our results and importance OA in wheat under stress. Moreover, osmotic adjustment contributed to YP through increasing the flag leaf area in wheat, as also reported by Farouk et al. [27]. On the other hand, a negative association between DSI with YP and yield components, including OA, indicates its status as a selection tool for drought-tolerant genotypes under DS conditions (Figure 4). Results provide insight into the role of OA in PY sustainability under the DS environment. High yielding genotypes under drought stress maintained higher OA having more TKW as compare to having less OA. Blue Silver, LLR-29, WC-4, WC-1, and WC-8 are the high yielding genotypes under DS environment holding less than 1 DSI_TKW_ and stand as drought-tolerant genotypes. Genotypes including LLR-13, Sehar-06, LLR-39, WC-3, and Punjab-96 were drought susceptible genotypes holding higher DSI_TKW_ and fall far away from the trait’s vectors in biplot analysis.

## 4. Material and Methods

### 4.1. Plant Material and Experimental Conditions

A panel of 30 wheat genotypes from Pakistan were screened during this study. This panel included 14 varieties, 9 local landraces (LLR), 6 wild crosses (WC), and 1 advanced line (Supplementary Table S1). A field experiment in a randomized complete block design (RCBD) was set up in different environmental conditions of water regimes: WW (well-watered) and DS (drought stress). For WW field conditions, all genotypes with three replications were hand-planted in the field and provided with normal irrigations till maturity. For DS field condition, all genotypes were planted with three replications under an iron frame tunnel covered by polythene sheet used as rain shelter. A 0.5 m deep ditch was dug around the tunnel to prevent any seepage of rainwater. Drought stress was imposed by withholding irrigations at four critical stages of wheat according to [47,48] with slight modifications. DS was imposed before tillering stage (30–40 days after sowing (DAS)), at booting stage (120–130 DAS), after ear emergence and grain filling phases by withholding irrigation as compare to control. Two-meter-long rows were planted of each genotype in triplicate, maintaining an inter-row spacing of 30 cm for both water regimes. The sowing was completed on1st November of the growing year, to establish a uniform stand, 35–40 viable seeds were sowed with a small-plot grain drill for each row. To ensure adequate nutrition, standardized agronomic practices were followed.

### 4.2. Evaluation and Data Recording

Data were recorded at physiological maturity for an osmotic adjustment. The osmotic adjustment was measured by the rehydration method of [49] with few modifications according to CIMMYT Guide [50]. Watering was provided to both water regimes to ensure the saturation before sampling. The next day morning, sampling was done after this; the solute potential of the leaf sap was measured by osmometer. The OP (Osmotic potential) readings were obtained in mmol kg^−1^ unit, which was converted to Mega Pascal (MPa) according to the Van’t Hoff equation:

(1) $$\mathrm{OP}\;\left(\mathrm{MPa}\right)=\frac{-R\times T\times \mathrm{osmoter}\;\mathrm{reading}}{1000}$$

Here, T was the laboratory temperature (30 °C) and R was the gas constant (0.008314). The osmotic adjustment was calculated by formula:

(2) OA=OP non−stressed – OP stressed 

according to [51]. Plant height (PH) (measured from the base of the plant to spike tip excluding awns), spike length (SL) (the point where spike originates to the terminal portion of the last spikelet excluding awns), number of tillers^−1^ plant (NTL) (randomly selected plants from each genotype), number of spikelets^−1^ spike (NSLS) (randomly selected plants from each genotype). After harvest, number of grains^−1^ spike (NGS) (grains on 10 random spikes from each genotype), thousand kernel weight (TKW) (measured by weighing at least 500 kernels from each genotype twice) and grain—yield^−1^ plant (YP). Drought susceptibility index for TKW (DSI_TKW_) was calculated using formula:

(3) $$\mathrm{DSITKW}\;=\;\frac{1-\frac{Y}{Y_{P}}}{D}$$

where *Y* is TKW of genotype under DS condition, *Y_P_* is mean TKW of genotypes under WW condition, *D* (stress intensity):

(4) $$D\;=1-\frac{X}{X_{p}}$$

where *X* is the mean of *Y* of all genotypes and *X_P_* is the mean of *Y_P_* of all genotypes. Genotypes were rated tolerant (DSI_TKW_ ≤ 0.50), moderately tolerant (DSI_TKW_ > 0.50 ≤ 1.00) or tolerant (DSI_TKW_ > 1.00) to drought stress.

### 4.3. Statistical Analyses

Statistix 8.1 software (Analytical Software, Tallahassee, FL, USA) was used to run an analysis of variance (ANOVA). SAS version 9.4 (SAS Institute, Cary, NC, USA) was used to run ridge regression analysis to determine yield predictability of the studied traits under WW and DS conditions. While JMP^®^ (Version (15.0), SAS Institute Inc., Cary, NC, USA, 1989–2019) was used to do principal component analysis (PCA) and other diversity analyses (plots and graphs in Figure 2 and Figure 4). Correlation analysis and graphing were carried out by using the corrplot package of R 3.0 software.

## 5. Conclusions

Genotypes were holding a significant variation for deliberated traits suggesting the importance of these candidate traits for the selection of drought-tolerant genotypes under DS conditions. Results interpret that OA and TKW are significantly associated with yield per plant having a direct and indirect effect on YP. Our study findings and previous reports comply that OA is a prime adaptive engine for drought tolerance in support of plant yield. We identified Blue Silver, LLR-29, WC-4, WC-1, and WC-8 comparatively high yielding genotypes under drought stress environment. A useful variation is present in the wheat genotypes which can be used for genome-wide association studies to identify the genomic regions for these important drought tolerant indicators. Additionally, the way of OA assists to maintain the plant growth and yield, its effecters, pathways, and genetic bases can be identified in future studies.

## Figures and Tables

**Table 1:** Evidence of osmotic adjustment (OA) association with plant productivity and yield.

Crop	Effects of OA on Drought Tolerance and Yield	Reference
Wheat	Potassium, glycine betaine and proline contributors to the leaf OA under DS condition and cumulatively assist to the grain yield	[14]
Wheat	Osmotic adjustment showed positive direct effects on shoot length, root length, fresh root weight, sugar and glycine betaine under drought stress	[15]
Wheat	Plants with better OA capacity and high benzoxazinone content have better field yields	[11]
Wheat	Overall, high osmoregulation increases grain yields in response to osmotic stress	[16]
Wheat	Osmotic adjustment sustained turgor maintenance and hence the yield-forming processes during moderate and severe water stress	[17]
Wheat	Indications of OA exist among wheat cultivars and associated with plant production under drought stress	[18]
Wheat and barley	Higher OA was found in genotypes exhibiting high yield stability across contrasting environments. Additionally, relative water content, leaf osmotic potential, and accumulation of soluble sugars were found to be highly related to osmotic adjustment.	[19]
Wheat	OA associated with the yield stability during the grain filling and ear growth under the DS condition	[20]
Wheat	Plants with higher osmoregulation extract more water from the soil and produce more dry matter and grain yield	[21]
Wheat	The yield of genotypes was 17% higher in bread wheat and 7% in durum wheat having higher OA	[21]
Wheat	Osmotic adjustment, water use efficiency WUE, and tissue elasticity are selection tools for the improvement of wheat drought tolerance	[22]
Wheat	The relationships suggested that direct selection for OA it may increase or decrease yield under drought but it depends on stress intensity	[23]

**Table 2:** Analysis of variance (MS values) for all the traits under WW and DS conditions.

SOV	DF	OA	PH	SL	NTL	NSLS	NGS	TKW	YP
Treatment	2	0.43 **	1130.02 **	169.80 **	84.54 **	146.95 **	529.78 **	917.28 **	449.28 **
Genotype	29	0.04 **	1433.06 **	7.51 **	6.18 **	10.95 **	327.63 **	73.64 **	8.93 **
G x T	58	0.03 **	97.65 ^NS^	2.53 **	0.88 **	4.14	80.75 *	48.80 **	3.92 **
Error	178	0.002	76.93	0.79	0.55	2.77	15.68	9.61	2.25

NS = non-significant, * = Significant and ** = Significant at the 0.05.

**Table 3:** Summary statistics and principal component squared cosines values (last 3 columns) for all traits.

Variable	Minimum	Maximum	Mean	Std. Deviation	F1	F2	F3
OA	0.4057	0.9190	0.6841	0.1258	0.1299	0.3386	0.4093
NTL	2.8222	9.5167	5.1142	1.3146	0.5478	0.1639	0.0078
PH	83.5444	140.3778	107.7956	13.7760	0.1402	0.1125	0.6336
SL	4.9083	13.5083	10.7098	1.7890	0.7571	0.0379	0.0274
NSLS	14.7667	22.1667	18.2166	1.9255	0.5950	0.0192	0.0197
NGS	22.4889	54.4889	40.3834	7.2232	0.2302	0.5697	0.0026
TKW	20.6670	47.6967	33.8196	5.3032	0.4800	0.0270	0.0244
YP	3.6333	12.2356	8.0751	2.5313	0.8717	0.0157	0.0014

Here, OA for osmotic adjustment; PH, plant height; NTL, number of tillers; SL, spike length; NSLS, number of spikelets per spike NGS, number of grains per spike; TKW, thousand kernel weight and YP Yield per plant.

**Table 4:** Thousand kernel weight (TKW) variability estimation model obtained by ridge regression along with estimated regression and R square values.

Environment	R.P Value	NGS	NTL	PH	SL	NSLS	YP	OA	R^2^
WW	0.3	0.065651	−0.91773	0.075970	0.391445	−1.10072	0.91381	10.4632	37.69%
DS	0.4	0.054466	−0.81762	0.070063	0.356197	−0.979303	0.878935	10.1628	54.54%

R.P Value = Ridge parameter value, R^2^ = R—Squared values in percentage.

## Supplementary Materials

The following are available online at https://www.mdpi.com/2223-7747/9/9/1208/s1, Figure S1: Biplot (Principal component analysis) for all traits under WW and DS conditions. Figure S2 Summary of biplot (Principal component analysis) for all traits under WW and DS conditions. Figure S3: Ridge traces of standardized regression coefficients for increasing values of ridge parameter, for osmotic adjustment and yield components, for drought stress (DS) and well-watered (WW) respectively. Table S1: List of 30 wheat genotypes evaluated in this study. Table S2: Mean performance of 30 wheats genotypes under WW and DS environment along with heat susceptibility index for yield per plant (HSI_YP_). Table S3: Cubic clustering criterion.
