# Supplementary material for: Incredible Role of Osmotic Adjustment in Grain Yield Sustainability under Water Scarcity Conditions in Wheat (Triticum aestivum L.)
Source: Plants (Basel). 2020 Sep 15;9(9):1208. doi: 10.3390/plants9091208 (PMC7569908; doi:10.3390/plants9091208)
Supplement: Supplementary file 1 [file plants-09-01208-s001.pdf]

Supplementary Figures

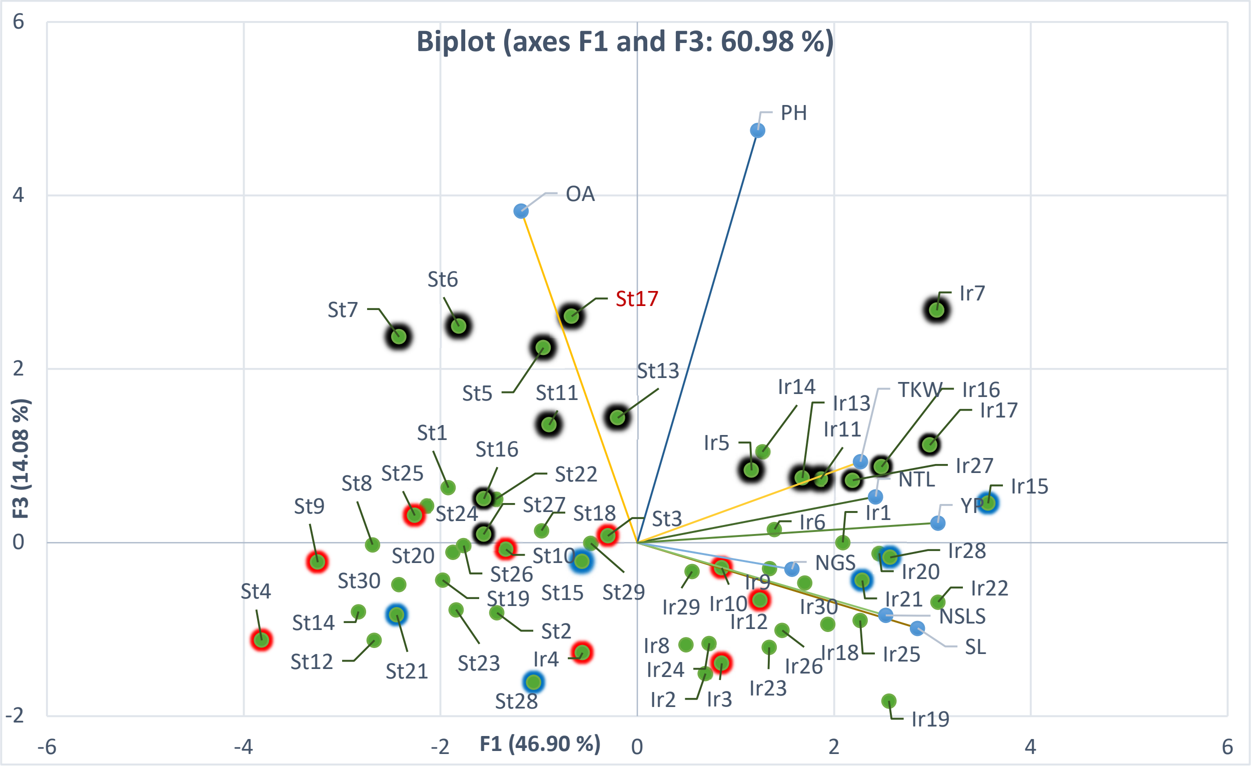

Figure S1. Biplot (Principal component analysis) for all traits under WW and DS conditions. Scatter-plot showed drought-tolerant, moderate and susceptible wheat genotypes for OA, yield and yield components according to first (F1) and third (F3) principal components. Here Ir1 to Ir30 represents genotypes under irrigation and St1 to St30 under stress conditions. For trait variables OA, osmotic adjustment; PH, plant height; NTL, number of tillers; SL, spike length; NSLS, number of spikelet's per spike NGS, number of grains per spike; TKW, thousand kernel weight and YP, yield per plant.

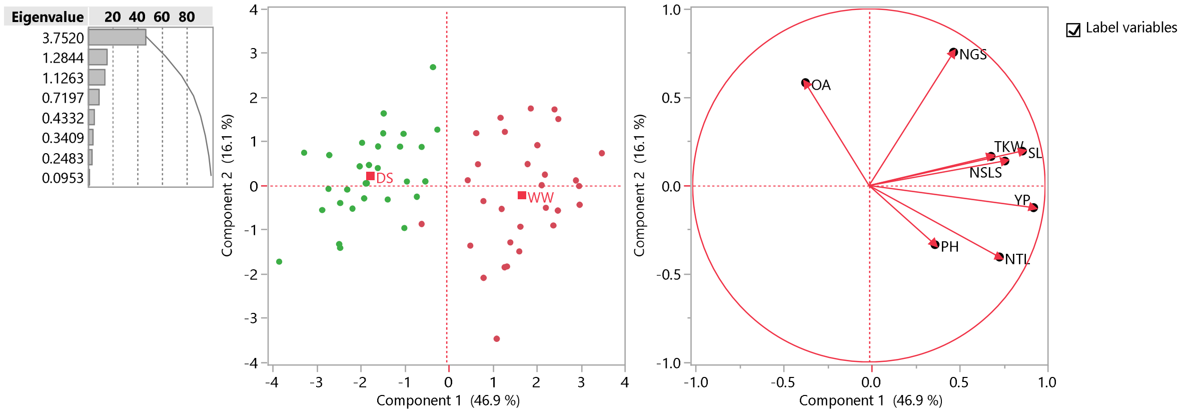

Figure 2. Summary of biplot (Principal component analysis) for all traits under WW and DS conditions.

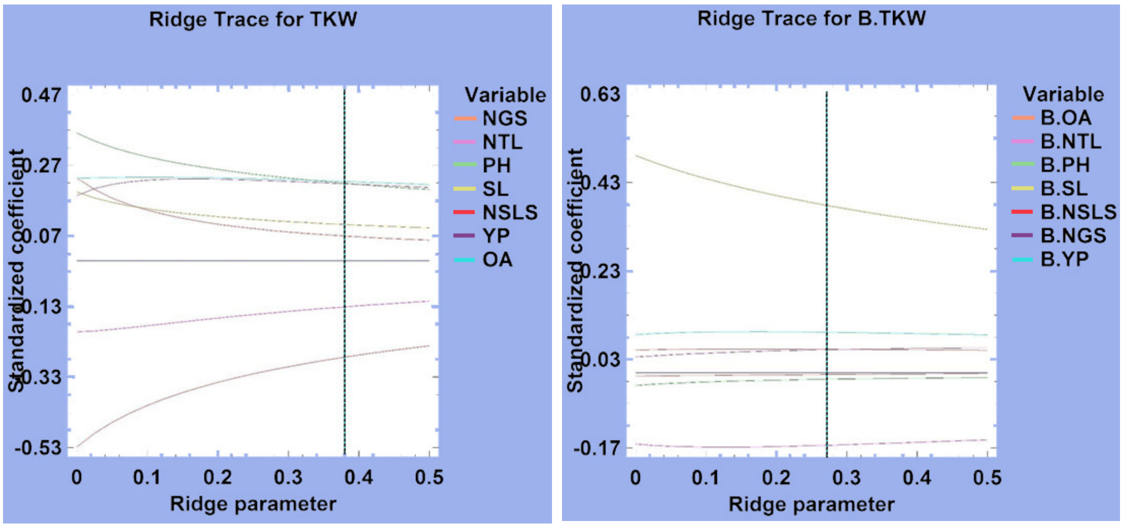

Figure 3. Ridge traces of standardized regression coefficients for increasing values of ridge parameter, for osmotic adjustment and yield components, for drought stress (DS) and well-watered (WW) respectively. Here OA, osmotic adjustment; PH, plant height; NTL, number of tillers; SL, spike length; NSLS, number of spikelet's per spike NGS, number of grains per spike; TKW, thousand kernel weight and YP Yield per plant.

Supplementary Tables

Table S1. List of 30 wheat genotypes evaluated in this study.

Sr. No.	Genotypes Name
**1**	C-271
**2**	C-273
**3**	WC-1
**4**	WC-3
**5**	WC-4
**6**	WC-8
**7**	WC-22
**8**	WC-25
**9**	LLR-4
**10**	LLR-13
**11**	LLR-14
**12**	LLR-19
**13**	LLR-29
**14**	LLR-39
**15**	Shahkar-95
**16**	Lasani-08
**17**	Blue Silver
**18**	MH-97
**19**	Maxi-pak
**20**	Fsd-83
**21**	Punjab-96
**22**	Kohsar-95
**23**	Lylpur-73
**24**	Auqab-2000
**25**	Sehar-06
**26**	Fsd-85
**27**	Pasban-90
**28**	Kohistan-97
**29**	AUR-0809
**30**	Dhrabi

Here, C for cultivar; WC, wiled cross; LLR, local land race; and others are the certified varieties of Pakistan.

Table 2. Mean performance of 30 wheats genotypes under WW and DS environment along with heat susceptibility index for yield per plant (HSI_{YP}).

Geno	DSI	OA	NTL		PH		SL		NSLS		NGS		TKW		YP	
		WW/DS	WW	DS	WW	DS	WW	DS	WW	DS	WW	DS	WW	DS	WW	DS
29	0.18	0.52	5.13	4.77	110.63	105.96	12.62	11.35	18.97	17.63	26.91	38.27	37.01	32.00	7.98	7.38
3	0.53	0.67	5.60	4.21	95.14	94.66	11.19	11.14	19.77	17.57	39.54	54.49	34.36	35.67	9.37	7.26
2	0.57	0.54	4.88	2.96	86.39	96.71	11.46	10.30	19.97	19.32	45.88	47.43	38.95	26.82	7.54	5.71
17	0.57	0.6	6.60	4.60	132.99	140.38	11.78	8.72	21.83	16.54	49.35	41.79	37.79	33.44	10.90	8.23
13	0.65	0.72	7.66	5.60	122.89	107.52	11.67	9.71	18.50	17.13	37.16	44.99	33.13	39.57	10.78	7.78
18	0.77	0.6	5.43	4.07	95.96	105.13	12.25	10.84	21.37	18.13	53.24	44.96	37.34	29.10	9.55	6.44
24	0.78	0.53	4.82	4.29	98.88	110.02	12.75	8.69	16.03	16.60	47.43	34.57	38.48	28.40	8.59	5.73
15	0.80	0.56	5.88	4.35	123.10	104.99	13.51	11.09	22.17	20.13	47.71	42.91	47.70	27.39	9.68	6.37
23	0.82	0.54	7.46	5.18	105.13	89.10	11.68	9.55	20.77	16.79	34.04	37.79	27.68	26.73	9.58	6.22
5	0.83	0.54	7.10	4.91	135.92	138.52	10.46	8.67	20.37	16.79	22.49	35.96	32.06	34.98	9.56	6.19
8	0.85	0.57	5.29	3.21	97.55	97.07	12.40	9.33	17.90	15.52	43.10	30.41	32.05	32.09	8.68	5.55
12	0.97	0.52	4.71	3.82	100.14	83.54	12.58	8.42	19.23	15.99	51.57	41.71	34.92	27.05	9.83	5.77
20	0.97	0.52	6.49	4.21	115.41	99.13	12.48	9.60	18.90	15.57	39.96	33.96	40.49	31.60	11.77	6.90
6	0.99	0.67	5.66	3.41	130.82	127.18	11.59	8.07	17.37	15.57	39.02	33.66	34.62	41.32	10.62	6.15
22	0.99	0.7	6.60	4.29	105.20	97.85	13.20	10.50	20.90	16.68	39.29	40.24	42.15	31.59	12.11	7.01
26	1.00	0.54	5.91	3.77	105.41	99.57	12.53	9.52	15.50	15.85	38.82	39.41	39.94	32.97	11.46	6.60
11	1.01	0.62	6.27	4.93	118.00	114.41	11.55	9.10	17.43	17.10	51.41	36.82	37.48	38.09	11.79	6.73
30	1.12	0.42	6.41	4.57	106.02	101.46	11.88	8.66	19.90	15.99	34.74	28.85	39.25	28.55	10.10	5.28
4	1.14	0.34	7.13	4.66	92.56	96.45	7.41	4.91	18.63	16.77	43.57	33.02	28.58	20.67	7.36	3.80
9	1.15	0.63	5.21	2.93	98.63	88.85	12.49	8.78	19.57	15.63	44.54	34.07	41.87	31.24	8.59	4.39
16	1.16	0.64	4.92	3.99	122.59	107.54	13.15	10.58	20.70	18.18	53.79	39.99	39.71	28.18	10.35	5.22
27	1.17	0.57	6.13	4.29	119.93	102.71	12.05	9.59	20.23	17.63	43.52	39.10	35.33	29.43	12.24	6.16
19	1.17	0.45	5.71	2.82	99.88	98.35	12.66	9.08	20.90	16.13	40.93	37.93	42.70	38.08	10.17	5.09
1	1.20	0.67	6.07	3.18	109.59	105.54	12.81	10.06	20.57	17.88	48.99	36.79	34.24	31.73	10.66	5.21
28	1.20	0.41	5.38	3.93	104.02	91.65	12.98	10.41	20.70	19.10	50.32	46.54	40.45	26.97	11.85	5.77
21	1.21	0.39	5.96	5.13	120.11	97.66	12.88	9.10	20.63	14.77	45.27	32.82	31.70	26.08	10.95	5.31
7	1.24	0.7	9.52	4.10	135.07	130.32	12.05	8.18	18.63	16.85	49.82	32.99	36.88	27.21	11.76	5.53
10	1.30	0.44	5.49	4.21	108.77	110.46	11.46	10.66	17.43	16.24	25.16	37.68	38.20	31.83	11.93	5.33
14	1.51	0.47	7.27	4.57	114.91	91.35	11.22	8.30	19.37	16.13	37.46	33.02	32.14	28.40	11.09	3.96
25	1.57	0.49	5.82	3.38	107.93	114.10	12.32	8.64	20.57	17.99	48.96	36.82	35.18	29.65	11.00	3.63

Here, OA for osmotic adjustment; PH, plant height; NTL, number of tillers; SL, spike length; NSLS, number of spikelets per spike NGS, number of grains per spike; TKW, thousand kernel weight and YP Yield per plant. WW for well-watered and DS, drought stress.

Table 3. Cubic Clustering Criterion.

	Number of Clusters	CCC	
	1	0.000	
->	2	0.462	
	3	-1.159	
	4	-2.242	
	5	-2.701	
	6	-2.694	
